# Microbial Art Fixation on Agar Plate: MicArt Fixation for Microbiology Teaching

**DOI:** 10.1002/jobm.70018

**Published:** 2025-03-06

**Authors:** Hatice Nur Halipci Topsakal, Murat Can Asarkaya, Ecem Akcan, Melih Çifçi, Rojda Karaman, Sudenaz Doğan, Elif Nur Çelik, Betül Bakir, Yasemin Günter, Okan Aydoğan

**Affiliations:** ^1^ Medical Laboratory Techniques Program, Vocational School Istanbul Atlas University Istanbul Turkey; ^2^ Molecular Biology and Genetics Department, Faculty of Engineering and Natural Sciences Istanbul Atlas University Istanbul Turkey; ^3^ Software Engineering Department, Faculty of Engineering and Natural Sciences Istanbul Atlas University Istanbul Turkey; ^4^ Computer Technology Program, Vocational School Istanbul Atlas University Istanbul Turkey; ^5^ Medical Microbiology Department Istanbul Medipol University School of Medicine Istanbul Turkey

**Keywords:** agar art, fixation, media, microbiology education

## Abstract

Art based science materials engage students for learning all of the unseen microscobic creatures in easy way microbiology field. In this study we aimed to visualize bacterial and fungal species in artistic way. Inoculation of the colourful strains on agar plate as painting the agar with bacteria, provides beautiful round scenes and clear reminiscence related with the species learning for microbe world. For this purpose, we determined the different concentrations and exposure times of formaldehyde, glutaraldehyde, xylene, toluene, ethyl alcohol, epoxy, glycerin and phenol. We aimed to develop a protocol after determining the optimum storage times. As a method, we exposed all chemicals except epoxy to Petri dishes by spraying method for 1, 5, 10 min. As a result, we determined the fixative that provides the best protection is toluene. After 1 min of toluene exposure, we determined that the Petri dish with growth can be preserved for 6 months even under room conditions without disturbing the color and shape of the whole colony morphology. Here, we obtained best fixation process by using toluen, typically 1 min, depending on the species, was most promising for the preservation of art.

**Abbreviations**
ASM: American Society for Microbiology
FEMS: Federation of European Microbiological Societies
IMD: International Microorganism Day
TMC: Turkish Microbiology Society
w/v: weight/volume

## 1 Introduction

Understanding the microcosmos can be complex in terms of recognizing microorganisms, learning about the diseases they cause, identifying their uses in biotechnology or know their place in normal flora. Classification and identification of microorganisms can be time‐consuming and sometimes exhausting for people in fields that require microbiology as a course [1]. Health professionals who need to know medical microbiology, engineering students who will use microorganisms in biotechnology, health or food technicians who will be personally involved in asepsis‐antisepsis applications must first acquire theoretical knowledge to learn microbiology and then be equipped with practical gains to varying degrees. Microscopes based on a lens system that makes microorganisms that are too small to be seen with the naked eye visible are very useful both for research in various fields and for training in microbiology courses. Today, microscope images are frequently used both in theoretical lectures and laboratory practices [2]. Different teaching methods are needed to prevent the loss of time experienced in finding a microscope field, to reduce image changes caused by microsetting, and to eliminate the necessity of creating a program that will have one microscope per student in crowded student groups [3]. Agar art is a form of bio‐art in which images are created by growing different species of bacteria or fungi on a solid growth medium. Agar artists effectively use bacteria instead of paint and agar instead of canvas. The first recorded agar artist was actually famous: the microbiologist Sir Alexander Fleming. Alexander Fleming taught himself to paint with watercolors, eventually taking this hobby to the lab and creating portraits of mothers, wrestlers and others using bacteria in Petri dishes. This creative endeavor may have led him to his experiments with what he then called “*Physarum polycephalum* (slime fungus)”, but which would later be called *Penicillium* and lead to a discovery that would change the course of history [4].

Inoculation of the colourful strains on agar plate as painting the agar with bacteria, provides beautiful round scenes and clear reminiscence related with the species learning for microbe world. After inoculation of the strains in artistic coreography, the fixation process of the Petri dishes must be completed. The microscopic field of the certain bacteria must be correlated with their artistic shape thus learners of the microbiology from each level‐beginner, intermediate or advanced microbiology‐ can integrate i) colony morphology, ii) microscopic field and iii) artistic shape. Correspondingly, the art of the bacteria has been composed on the agar then the general microbiological categorization have been drawn and the items have been converted to education materials for microbiology learners. Thus the science and art will not be only integrated but also complete to each other [3].

The American Society for Microbiology (ASM) has produced some visual materials on how to create agar art with living microbes. It has also organized an annual photo contest to select the best Agar Art [4, 5] since 2015. Similarly, International Microorganism Day (IMD) is celebrated all over the world under the leadership of the Federation of European Microbiological Societies (FEMS) and competitions involving agar art are organized regularly every year. Similarly, the Turkish Microbiology Society (TMC) occasionally includes agar art workshops in its annual Turkish Microbiology Congress, activities or competitions, the 41st of which was organized this year.

Bacterial art, which can also be considered as a living art, is based on the artistic forms of Petri dishes created using bacterial strains of different colors. Agar art, which can be preferred to make microbiology learning fun, is related to the principle of bringing both science and art together. This way of learning will also help in teaching and recognizing human‐associated microorganisms and categorizing them with knowledge about their pathogenicity. Part of the problem in producing agar art over a period of time as a result of the product is the use of living organisms. This leads to the loss of part of the original work, and the only way to preserve the image and keep a record of the work is to photograph it. There are very few studies on fixation of microbes grown on agar because most preservation studies involve fixation of tissue sections or cells grown on a surface as part of a tissue culture study [5, 6].

**Figure 1:**
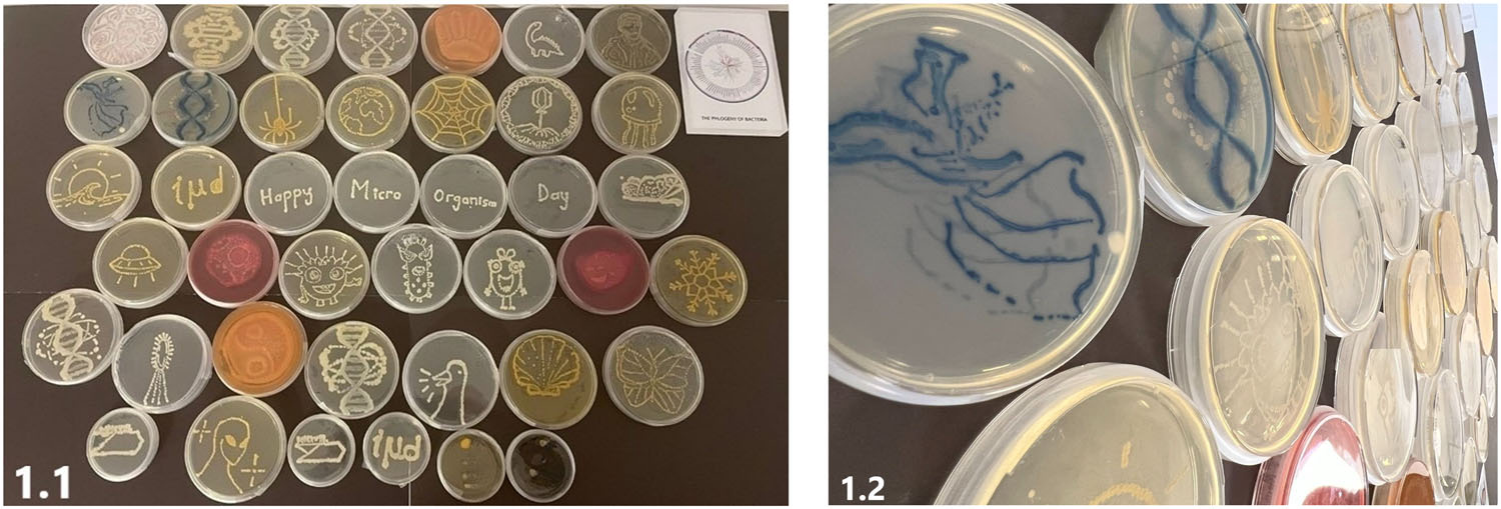
Petri plates inoculated with live bacteria containing agar art prepared for the SCI‐ART corner we organized on our university campus as part of the International Microorganism Day (IMD) celebrations.

The aim of this study was to evaluate and compare the most commonly preferred different chemicals used for fixation and different fixation times to preserve agar artworks for use in teaching microbiology. Thus, the chemicals and application times that would ensure optimum fixation were investigated, and after the time and concentration ratios were determined, the selected protocol was deemed appropriate for use in agar art production and storage. With the developed protocol, it will be possible to transform the Petri dishes to be used in microbiology practices into works of art and the Petri dishes can be used for 6 months. Since the designed Petri dishes will have artistic visual designs, they will not only attract the attention of the trainees, but will also enable them to learn microorganisms more easily and effectively [7]. The designed and fixed Petri dishes will also be ready to be used for SCI‐ART exhibitions or fairs to be set up at different times and places (see Figure 1).

## 2 Materials and Methods

In the study, we aimed to perform bacterial art by inoculating naturally colored strains on selective and general media. We planned to grow the natural colonies on Petri plates containing medium and present their colony shapes and colors in a striking way.

While learning the names and shapes of species other than natural colony appearances by making different drawings; we thought that striking appearances of colors and shapes would facilitate reminding and make learning enjoyable. We aimed to select a fixative that can be stored for 6 months for the exhibition of the design products created and to develop a protocol [8]. For fixative selection, we used an average of 3 different concentrations of 8 different chemicals such as formaldehyde, glutaraldehyde, xylene, toluene, ethyl alcohol, epoxy, glycerin and phenol. The brand names and concentration rates of the chemicals have shown in Table 1.

**Table 1:** Concentrations of chemical materials used for fixation and exposure times to cultured Petri dishes [9, 10].

	Fixatives	Concentrations	Exposure time	Brand and quantity
1	Formaldehyde	1%; 2%; 3.7%	1 min; 5 min; 10 min	Rokim, 1 Lt
2	Glutaraldehyde	1%; 2.5%; 100%	2 min; 5 min	Sigma Aldrich, 1 Lt
3	Xylene	2.0%; 3.7%; 100%	1 min; 5 min; 10 min	Rokim, 1 Lt
4	Toluen	1%; 2.5%	1 min; 5 min; 10 min	Kimetsan, 1 Lt
5	Ethyl alcohol	2.0%; 3.7%	1 min; 5 min; 10 min	Rokim, 1 Lt
6	Epoxy	100%	1 min; 5 min; 10 min	Transparent Epoxy, 75 g
7	Glycerin	1.0%; 2.0%; 3.7%	1 min; 5 min; 10 min	Sigma Aldrich, 1 Lt
8	Phenol	1%; 2.5%; 100%	1 min; 5 min; 10 min	Liquid, Kimetsan 1 Lt

### 2.1 Bacterial Strains to Be Used for Agar Art

*Escherichia coli, Staphylococcus aureus, Pseudomonas aeruginosa, Klebsiella pneumoniae, Enterobacter cloacae* bacterial species were cultured from stock cultures identified by the automatized identification system VITEK 2 GN cards (BioMérieux, Marcy l'Etoile, France) by cultivation. Baird Parker Agar, Mueller Hinton Agar, Chrome agar, Luria Bertani agar, Nutrient agar, Sheep blood agar (Sigma Aldrich, Germany) were selected as media.

During inoculation process, we followed those steps: the inoculation loop or swab was handled to the Petri dish containing the bacteria‐derived agar. It was moved it over the 1st quadrant of the agar plate using a reciprocating motion to spread the bacteria. Using a new sterile tip, continued the cycle, touched the inoculation loop to one edge of the quadrant and extended the lines to the 2nd quadrant and repeated for the 3rd and 4th quadrants using a reciprocating motion [11]. The Petri dishes were incubated at room temperature, upside down ‐agar side up‐ until the colony grows. A sterile toothpick or pipette tip was used for transfer. This process was repeated for all microorganism samples [12].

### 2.2 Agar Art Preparation

In agar art, a template was attached to the bottom of the Petri dish to ensure uniformity of the images drawn on a piece of paper. Drawings were made on the top of the Petri dish with an acetate pen. All agar art was performed using 9 cm Petri dishes containing 1.5% (w/v) nutrient. Media Baird Parker Agar, Mueller Hinton Agar, Chrome agar were prepared under appropriate conditions. The medium was stored at 4°C to prevent contamination before inoculation. One or at most two different bacteria were used for the same Petri and each field was used only once in each position. We selected 8 fixatives with different concentrations (Table 1). The Petri plate was used for all microbes growing simultaneously. Before any fixation step, images of the bacteria were taken. The dish was wrapped in parafilm to avoid drying out. All 6‐month observations were carried out under room temperature and humidity conditions. Petri dishes were stored upside down (agar side up) to avoid contamination and drying of the agar. Since the selected bacteria are mesophilic, incubation at 37°C for 24 h ensured sufficient growth. This period should be followed for a longer period of time into fastidious bacterial species [13, 14, 15]. Petri dishes with 1‐day growth were then subjected to fixation trials. A few examples of growth plates containing agar art are shown in Figure 1.

### 2.3 Fixation of Petri Plates by Exposure to Different Chemicals

Petri plates with different designs of agar art were exposed to different types and concentrations of chemicals and various retention times were tested. The chemicals and times used for fixation are given in Table 1. Exposure trials were carried out by spraying the Petri dishes in a vertical position at a distance of 5 cm from the spray bottle (paradise brand, 200 mL) at the specified time periods.

**Figure 2:**
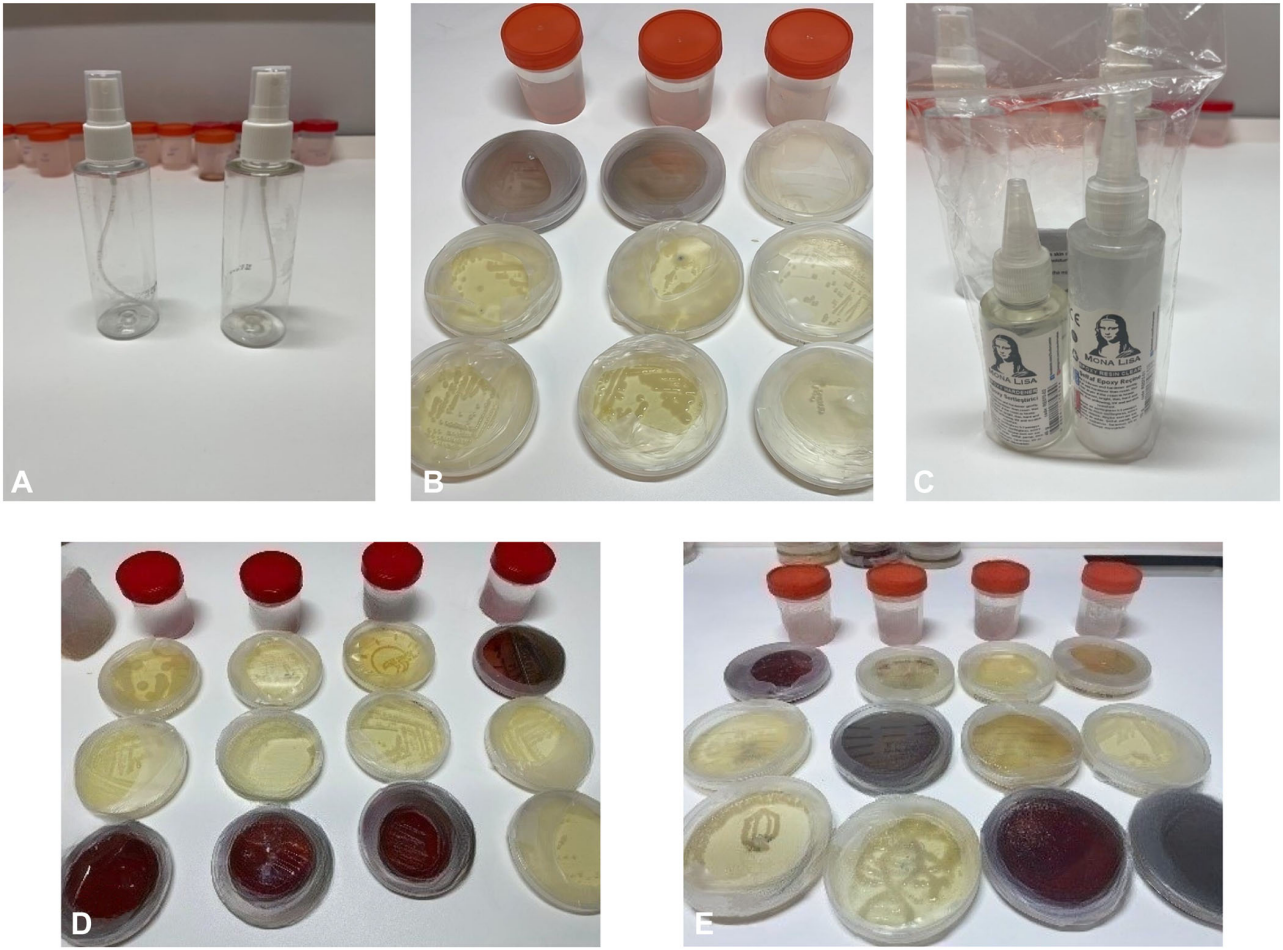
Spray bottles and plastic containers used for different concentrations of chemicals (A); Petri plates showing fixation experiments using different concentrations of xylene (B); Epoxy bottles (C); Petri plates showing fixation experiments using different concentrations of formol (D); Petri plates showing fixation experiments using different concentrations of glycerin (E).

One mL of each chemical was taken per Petri dish surface volume and transferred through a pipette. Among the chemical materials, only epoxy was not subjected to dilution by any dilution process since it has a dense resin consistency. Epoxy was applied to the Petri plates by dripping method in a volume of 1 mL at a concentration of 100% (Figure 2).

Petri dishes containing agar art with chemical exposure were observed for consecutive periods of 1 week, 2 weeks, 1 month, 3 months and 6 months and records were kept and images were taken. They were examined by 3 researchers in the categories shown in Table 2. Fixed appearance refers to the contents of the Petri dish stored without any deterioration in the reproductive Petri dish, while the contents of the Petri dish categorized as very well fixed refers to Petri dishes that are very well preserved in terms of both colony shapes and color visuals. For the chemical types and concentration ratios in the unfixed Petri dishes, descriptions with sub‐detailings as (i) contaminated appearance; (ii) melted appearance; (iii) yellowed appearance (iv) dried appearance were also used. Table 2 summarizes the descriptions used for the Petri dishes recorded during the observation.

**Table 2:** Evaluation legend.

Abbreviations	Meaning
F	Fixed
XX	Very well fixed, clear appearence
KONT	Contaminated
ERM	Melted appearence
SRM	Yellowish appearence
KR	Dried appearence

### 2.4 Statistical Analysis

According to the chi‐square test results, χ^2^ = 52.622; *p* = 0.001. The results are interpreted according to these two hypotheses: Hypothesis1. At least one or more chemicals achieved the appearance of fixation at level 1. Hypothesis 0: No chemicals were able to fix the Petri dishes and the results were always obtained at level 3.

## 3 Results

### 3.1 Views of Fixed Petri Plates

**Figure 3:**
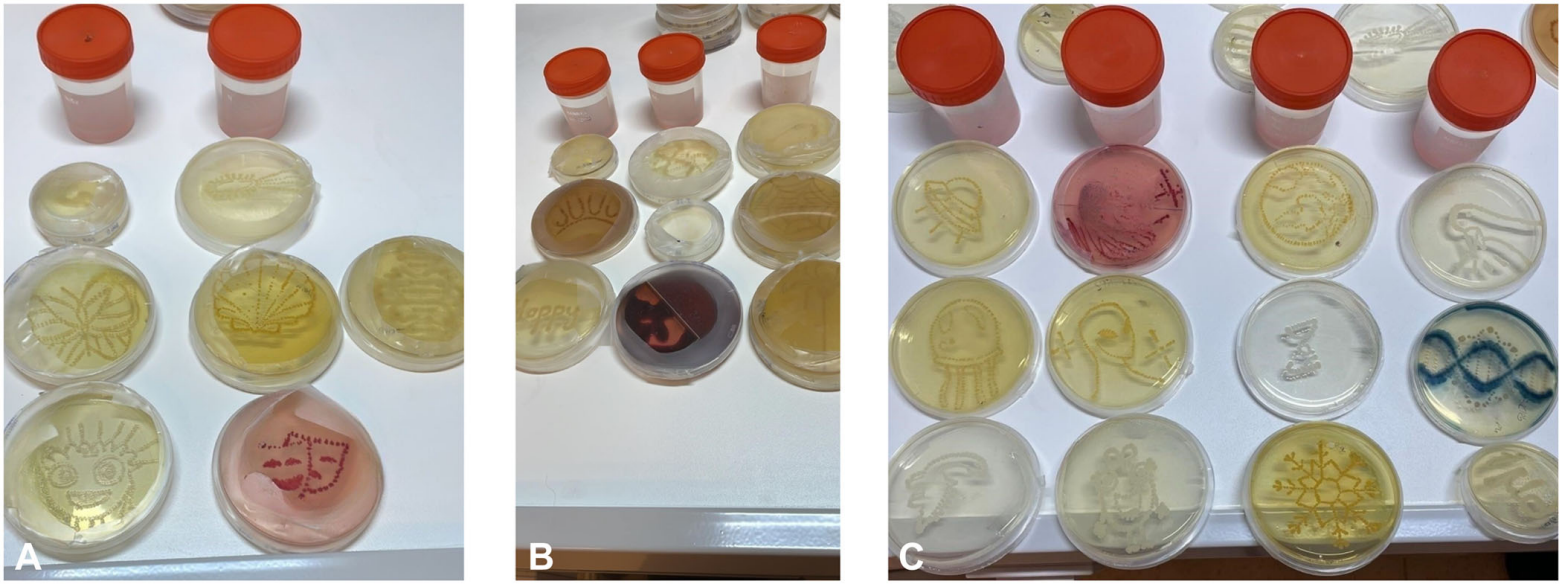
6‐month view of fixation using toluene, *E. coli* cultured on Mueller Hinton Agar (A); View 6‐month view of fixation using phenol, *E. coli* cultured on Mueller Hinton Agar (B); *E. coli* cultured on Mueller Hinton Agar, 6‐month view of fixation using ethyl alcohol (C).

The most successful fixation results were obtained with toluene, phenol and ethyl alcohol in fixation trials using 8 different chemicals. As a result of the trials, it was determined that the Petri dishes could remain stable in terms of color and structure for 6 months. The appearance of Petri plates fixed with toluene, phenol and ethyl alcohol for 6 months is shown in Figure 3 (Table S1).

### 3.2 Statistical Analysis Results

The null hypothesis Ho is rejected. According to this result, the relationship between chemical types and fixation appearance is significant at 95% confidence level. Hypothesis 1. At least one or more chemicals achieved fixation appearance at level 1.

8 chemical types were used on 337 Petri dishes, 88 of them were not fixed, 130 of them were moderately fixed and 119 of them were very well fixed. In other words, it can be said that 26.1% of the 337 Petri dishes in which 8 chemical types were used were not fixed, 38.6% were moderately fixed and 35.3% were very well fixed. Finally, Formol chemical type was used in 36 of 337 Petri dishes, gluteraldehyde chemical type in 36, xylene chemical type in 36, toluene chemical type in 46, ethyl alcohol in 65, Epoxy in 10, glycerin in 36 and phenol in 72. According to the chi‐square test results, X2 = 72,168 *p* = 0.001. According to this result, the relationship between chemical types and fixation appearance is significant at 95% confidence level.

According to the results of Kruskal‐Wallis H test statistic, H = 1.116 and *p* = 0.993. (p>α), there is no statistically significant difference between chemical types on fixation observation time so, it was chosen the accurate one as 1 min for toluene. Chemicals exposure times (1 min, 5 min, 15 min) did not effect the fixation results so the most rapid exposure time, 1 min, has been chosen for best fixation.

According to the results of the Kruskal‐Wallis H test statistic, the H = 72.511 and the *p* = 0.001 (p<α), There is a statistically significant difference between chemicals on concentration at 95% confidence level. The findings indicate a statistically significant correlation between the types of chemicals and the occurrence of fixation, with a 95% confidence level.

## 4 Discussion

Alexander Fleming used different bacteria from a wide variety of colors to obtain different colors [16]. However, the microbes that make up artworks have an even longer term in history; for example, the Bradshaw Rock artwork in Australia retains its vitality even though none of the original paint remains. This is because microorganisms grow in the area where the ink was, preserving the contours of the engraving and creating a microenvironment that allows microorganisms to grow [17].

It is also known that microorganisms have effects on historical artworks. For example, it is observed that artworks from the Renaissance period were influenced by microorganisms [18]. We wondered whether agar art would be useful not only in public space but also in the microbiology classroom. In the study, it was found that just watching the agar art exhibition stimulated the visitors' curiosity about basic microbiology concepts at a basic level and facilitated their understanding of the subject [19]. In our study, it was understood that Petri plates containing agar art in different designs, in which microorganisms are used as a color palette, can be stored for a long time and these materials, which are interesting in terms of color and content, can be used in microbiology teaching.

Fixative methods are divided into several groups and one or more of the chemical groups can be used: Alcohols, oxidizing agents or metal agents. Unfortunately, no single fixative can provide the desired effect for all specimens, so different fixatives are used for each specimen [20, 21]. Therefore, it is necessary to check the effects of different fixatives for different purposes and the working order after fixation [22]. This applies to histological sections as well as agar plates containing microbial colonies.

The temperature at which the material is fixed is important, it can provide morphologic preservation by increasing the rate at which the chemical reaction occurs, but this can also mean that it can accelerate the degeneration of any material [23]. Although fixation is an attempt to prevent tissue degradation by microorganisms during storage, it is also recognized that most fixation methods will lead to some changes in the tissue, such as shrinkage or hardening. Therefore, it is important to investigate the different factors that may affect the efficacy and rate of fixative [10].

In the study, we found that toluene (54.3%), ethyl alcohol (47.7%), and phenol (47.2%) were the chemicals that gave the highest rate of fixation among 8 different chemicals. All observations were carried out at 25°C, especially since it is thought that the principle of storage under room conditions will facilitate Petri display or examination. Wilson et al. [4] used 1%, 2% and 3.7% formaldehyde, 1% and 2.5% glutaraldehyde in their study.

According to the results of this study, which is the first article written in the literature on agarart fixation. In literature; 2% formaldehyde gave good fixation results after 1 min exposure. According to our study, formol provided very good fixation at a rate of 27.8% and was behind the other 3 chemicals used as a percentage. Very few the agar art articles in the literature, numbering no more than 5, include information about fixation [24], so this situation shows that the results of our study are quite remarkable and important.

The limitation of the study is the lack of student comments, questionnaires, pre‐test or post‐test results, although information was provided that the fixed Petri plates could be used in exhibitions and education. The fixed Petri plates were presented to audiences at all levels ‐ high school, university ‐ during the week of International Microorganism Day and attracted considerable interest. However, this research should be developed for further trainings using the fixed Petri dishes and in addition, the results of the analysis should be added to another study and presented to the literature to prove whether there is effective teaching or not.

## Author Contributions

**Hatice Nur Halipci Topsakal:** funding acquisition, validation, conceptualization, investigation, writing – original draft, writing – review and editing, visualization, supervision, resources, project administration, formal analysis. **Murat Can Asarkaya:** methodology. **Ecem Akcan:** methodology. **Melih Çifçi:** software. **Rojda Karaman:** methodology. **Sudenaz Doğan:** methodology. **Elif Nur Çelik:** methodology. **Betül Bakir:** methodology. **Yasemin Günter:** data curation, software, formal analysis. **Okan Aydoğan:** writing – original draft, writing – review and editing.

## Conflicts of Interest

The authors declare no conflicts of interests.

## Supporting information

Supporting information.

## Data Availability

The data that supports the findings of this study are available in the Supporting material of this article.
